# Supplementary material for: Causal Relationship between Aging and Anorexia Nervosa: A White-Matter-Microstructure-Mediated Mendelian Randomization Analysis
Source: Biomedicines. 2024 Aug 16;12(8):1874. doi: 10.3390/biomedicines12081874 (PMC11351342; doi:10.3390/biomedicines12081874)
Supplement: Supplementary file 1 [file biomedicines-12-01874-s001.zip › Supplementary Figure/Figure S4/Singe SNP plot of MR analysis for TL to ubm-b-1861.pdf]

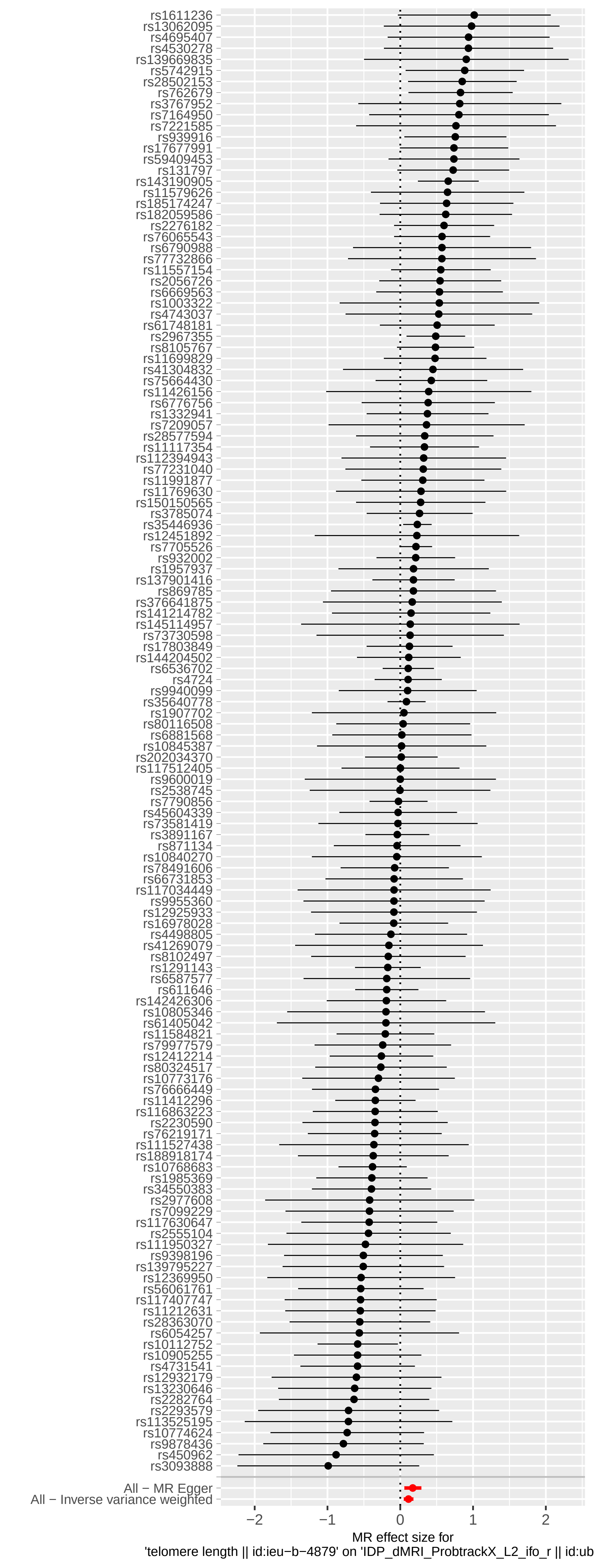
rs1611236
rs13062095
rs4695407
rs4530278
rs139669835
rs5742915
rs28502153
rs762679
rs3767952
rs7164950
rs7221585
rs939916
rs17677991
rs59409453
rs131797
rs143190905
rs11579626
rs185174247
rs182059586
rs2276182
rs76065543
rs6790988
rs77732866
rs11557154
rs2056726
rs6669563
rs1003322
rs4743037
rs61748181
rs2967355
rs8105767
rs11699829
rs41304832
rs75664430
rs11426156
rs6776756
rs1332941
rs7209057
rs28577594
rs11117354
rs112394943
rs77231040
rs11991877
rs11769630
rs150150565
rs3785074
rs35446936
rs12451892
rs7705526
rs932002
rs1957937
rs137901416
rs869785
rs376641875
rs141214782
rs145114957
rs73730598
rs17803849
rs144204502
rs6536702
rs4724
rs9940099
rs35640778
rs1907702
rs80116508
rs6881568
rs10845387
rs202034370
rs117512405
rs9600019
rs2538745
rs7790856
rs45604339
rs73581419
rs3891167
rs871134
rs10840270
rs78491606
rs66731853
rs117034449
rs9955360
rs12925933
rs16978028
rs4498805
rs41269079
rs8102497
rs1291143
rs6587577
rs611646
rs142426306
rs10805346
rs61405042
rs11584821
rs79977579
rs12412214
rs80324517
rs10773176
rs76666449
rs11412296
rs116863223
rs2230590
rs76219171
rs111527438
rs188918174
rs10768683
rs1985369
rs34550383
rs2977608
rs7099229
rs117630647
rs2555104
rs111950327
rs9398196
rs139795227
rs12369950
rs56061761
rs117407747
rs11212631
rs28363070
rs6054257
rs10112752
rs10905255
rs4731541
rs12932179
rs13230646
rs2282764
rs2293579
rs113525195
rs10774624
rs9878436
rs450962
rs3093888
All − MR Egger
All − Inverse variance weighted
−2
−1
0
1
2
MR effect size for
'telomere length || id:ieu−b−4879' on 'IDP_dMRI_ProbtrackX_L2_ifo_r || id:ub
